# Whole-Body and Segmental Phase Angles and Cognitive Function in the Older Korean Population: Cross-Sectional Analysis

**DOI:** 10.2196/63457

**Published:** 2024-12-16

**Authors:** Jiaren Chen, Jong-Hwan Park, Chien-Yu Lin, Ting-Fu Lai, Du-Ri Kim, Myung-Jun Shin, Eunsoo Moon, Jung Mo Kang, Jong Won Lee, Yoon Jae Cho, Yung Liao, Tae Sik Goh, Jung Sub Lee

**Affiliations:** 1 Graduate Institute of Sport, Leisure and Hospitality Management, National Taiwan Normal University, Taipei, Taiwan; 2 Department of Convergence Medicine, Pusan National University School of Medicine, Yangsan, Republic of Korea; 3 Department of Clinical Bio-Convergence, Graduate School of Convergence in Biomedical Science, Pusan National University School of Medicine, Yangsan, Republic of Korea; 4 Convergence Medical Institute of Technology, Pusan National University Hospital, Busan, Republic of Korea; 5 Department of Public Health, College of Public Health, China Medical University, Taichung, Taiwan; 6 Faculty of Sport Sciences, Waseda University, Tokorozawa, Japan; 7 Centre for Urban Transitions, Swinburne University of Technology, Melbourne, Australia; 8 Biomedical Research Institute, Pusan National University Hospital, 179 Gudeok-ro, Seo-gu, Busan, 49241, Republic of Korea; 9 Department of Rehabilitation Science, Graduate School, Inje University, Gimhae, Republic of Korea; 10 Department of Rehabilitation Medicine, Pusan National University School of Medicine, Pusan National University Hospital, Busan, Republic of Korea; 11 Department of Rehabilitation Medicine, Pusan National University School of Medicine, Yangsan, Republic of Korea; 12 Department of Psychiatry, Pusan National University Hospital, Busan, Republic of Korea; 13 Department of Psychiatry, Pusan National University School of Medicine, Yangsan, Republic of Korea; 14 Department of Orthopaedic Surgery, Pusan National University Hospital, 179 Gudeok-ro, Seo-gu, Busan, Republic of Korea, Busan, 49241, Republic of Korea; 15 Department of Orthopaedic Surgery, Pusan National University School of Medicine, 49 Busandaehak-ro, Mulgeum-eup, Yangsan, 50612, Republic of Korea, 82 51-240-7949, 82 51-247-8395

**Keywords:** bioelectrical impedance analysis, oxidative stress, cellular health, cognitive function, older adults, BIA, phase angle, PhA

## Abstract

**Background:**

Recently, the phase angle (PhA) has emerged as an essential indicator of cellular health. Most studies have examined its association with physiological conditions, such as sarcopenia, frailty, and physical function, in older populations. Simultaneously, growing attention is being paid to the clinical relevance of segmental PhAs for future applications. However, few studies have explored the relationship between PhAs, especially segmental PhAs, and the psychological aspects of health, particularly cognitive function.

**Objective:**

We aimed to investigate the association between whole-body and segmental PhAs and cognitive function in older adults.

**Methods:**

Individuals aged 65 years and above were recruited from adult community groups residing in Busan, South Korea, through the 2022 Bus-based Screening and Assessment Network (BUSAN) study of Pusan National University Hospital. Participants’ whole-body and segmental PhAs were measured using a bioelectrical impedance analyzer (BWA 2.0 Body Water Analyzer, InBody), and cognitive functions (overall and subdomains, including memory, orientation, attention and calculation, and language) were self-reported using the Korean version of the Mini-Mental State Examination. Multiple linear regression analyses were performed to examine these associations.

**Results:**

This study included 625 older adults aged 65‐96 years (women: n=444, 71%; men: n=191, 29%). A positive association was observed between whole-body PhA and cognitive function (*b*=0.62, 95% CI 0.16‐1.08; *P*<.01). We observed significant positive associations between the PhA of the lower limbs (*b*=0.72, 95% CI 0.38‐1.06; *P*<.001) and cognitive function. Analysis of the Mini-Mental State Examination subdomains revealed that whole-body PhA was significantly related to memory (*b*=0.11, 95% CI 0.00‐0.22; *P*=.04); the PhA of the upper limbs was significantly related to orientation (*b*=0.29, 95% CI 0.09‐0.49; *P*=.01); and the PhA of the lower limbs was significantly related to orientation (*b*=0.24, 95% CI 0.10‐0.38; *P*<.001), attention and calculation (*b*=0.21, 95% CI 0.06‐0.37; *P*=.01), memory (*b*=0.14, 95% CI 0.05‐0.22; *P*=.001), and language functions (*b*=0.07, 95% CI 0.01‐0.12; *P*=.01). However, trunk PhA showed no significant association.

**Conclusions:**

Our findings bolster the emerging evidence of a significant positive correlation between whole-body PhA and cognitive function in our sample, with nuanced relationships observed across different segmental PhAs and cognitive subdomains. Therefore, this study revealed that PhAs could be a useful tool for screening or preventing cognitive decline in the general older population, offering substantial evidence for future interventional studies. Further research should delve into the mechanisms and assess targeted interventions that enhance regional physical function to support cognitive health in older adults. Further long-term investigation on these associations is warranted.

## Introduction

The global population is aging, and this makes older adults’ well-being a primary public issue. Cognitive functioning declines with age, increasing the risk of dementia; prevalence rates have notably risen by 18% to 38% among those aged 85 years and above [1,2]. Dementia is the main cause of dependency and disability among older adults [3]. Cognitive decline appears to be irreversible once dementia is diagnosed. Therefore, there is an urgent need to identify measures to prevent cognitive decline [4]. Improving or maintaining cognitive health in older populations is important not only for individual well-being but also for societal sustainability [5]. Hence, maintaining cognitive health is not merely a means to diminish the risk of debilitating disorders, such as dementia, but it is also pivotal to facilitating better engagement in daily activities and enhancing social participation. Identifying biomarkers associated with cognitive function in the older population can be instrumental in enhancing our understanding of cognitive health and promoting healthy aging.

As individuals age, the accumulation of senescent cells increases, promoting proinflammatory cytokine secretion that disrupts normal cell and tissue function, negatively affecting cellular health and contributing to age-related diseases [6]. The phase angle (PhA) is emerging an indicator of muscle quality that reflects cellular health status [7] and is associated with inflammatory biomarkers [8,9]. Its values typically decline with age, indicating a loss of cellular membrane integrity [10]. A higher PhA is associated with lower risk of sarcopenia [11], frailty [12], and incident disability [13] as well as better physical function [14,15] in older adults. This may be attributed to higher PhAs indicating better muscle quality and mass. Furthermore, recent studies revealed a positive association between muscle health and cognitive functions [16,17]. However, few studies have explored the relationship between PhA and psychological conditions, such as cognitive function, in the older population [18].

Only 1 recent study has examined the association between PhA and clinically diagnosed Alzheimer disease or amnestic mild cognitive impairment in patients who visited an outpatient geriatrics department [19]. Thus far, few studies have addressed the relationship between PhA and nonclinically diagnosed cognitive impairment in a broader community-based older population [18,20]. Identifying subtle or atypical decline in cognitive function is crucial for early detection and intervention, and it is often overlooked in traditional clinical settings. Additionally, recent research suggests that segmental PhAs may serve as a prognostic tool for specific physical functions related to body segments [21,22]. Understanding the association between whole-body and segmental PhAs and cognitive function in older adults may aid in detailed health monitoring.

We investigated the associations between PhA, both whole-body and segmental, and cognitive functioning, overall and by subdomains, in older Korean adults.

## Methods

### Participants

Participants aged 65 years and above were selected from 18 different welfare centers and day care centers in Busan, South Korea, through the 2022 Bus-based Screening and Assessment Network (BUSAN) study of Pusan National University Hospital (July 1 to December 31, 2022). This program aimed at operating a specially modified medical bus to provide comprehensive health care services (including medical examinations, health consultations, and health education) to the older population by visiting public facilities in Busan. The target population was initially identified by the Busan Metropolitan City Health and Welfare Division, focusing on older individuals who may have difficulty accessing regular health care services. The welfare centers assisted in promoting the project and organizing the participation of older adults. A total of 1516 older individuals received health screening services. Subsequently, medical staff from the Health Promotion Center of Pusan National University Hospital, including specialists in orthopedics, nuclear medicine, and emergency medicine, conducted health checks and consultations for the selected participants. Each location was typically visited twice, allowing for follow-up assessments. These medical buses are equipped with mobile instruments, such as a bioelectrical impedance analyzer (BIA) and ultrasound equipment. Each participant filled in a questionnaire on sociodemographic characteristics and the Korean version of the Mini-Mental State Examination (K-MMSE) items on cognitive function; their PhA was objectively assessed using the instrument. All assessments were conducted in the morning, and participants were ensured a full rest and a regular diet to minimize the effects of fatigue or hunger. The tests were administered by trained health care professionals, following a standardized protocol in a controlled environment to ensure consistency across participants. Informed consent was obtained from all patients prior to data collection.

### Measurements

#### Phase Angle

Participants’ whole-body and segmental PhAs, such as the arms, trunk, and legs, were measured using a segmental multifrequency BIA (BWA 2.0 Body Water Analyzer, InBody), the reliability and validity of which have been extensively examined in previous research [23-25]. To maintain the measurement accuracy, the instrument was calibrated prior to each measurement. PhAs were assessed at a frequency of 50 kHz from raw resistance and reactance data, with higher values indicating greater integrity of the cell membranes [26]. The BIA used 4 electrodes that trained personnel placed on the participant’s hands and feet while the participant was seated for measurement. Participants with artificial implants in their bodies, such as cardiac pacemakers or metal orthopedic pins, or those with major limb amputations or significant sequelae from saphenous vein removal, which would impede the measurement of bioelectrical impedance [27], were excluded from the study.

#### Cognitive Function

Cognitive function was assessed by using the K-MMSE. It is a validated cognitive impairment screening tool widely employed for older adults. Its reliability and validity have been previously reported [28,29]. It evaluates cognitive functioning across 6 dimensions: orientation (10 points), attention and calculation (8 points), memory (3 points), language (5 points), verbal comprehension and functional capacity (3 points), and constructional praxis (1 point). The K-MMSE score, which ranges from 0 to 30, was calculated by summing the correct responses to each subitem of the assessment [30]. A higher score on overall cognitive functioning and its subdomains indicates better cognitive function.

#### Covariates

Covariates in the analysis included gender, age group (65‐74 years or ≥75 years), BMI (kg/m^2^), depressive symptoms, and nutritional status. Depressive symptoms were self-reported using the Korean version of the 15-item Geriatric Depression Scale, ranging from 0 (lowest risk) to 15 (highest risk) [31]. Scores of 5 or lower indicate no risk of depression, whereas scores of 6 or higher suggest the presence of depressive symptoms. Its reliability has been tested in the Korean older population, with a Cronbach α value of .86 and validity of .67 to .68 for screening depression [32]. Nutritional status was assessed using the Korean version of the Mini Nutritional Assessment Short Form, which has been validated in previous studies in older populations [33]. The assessment comprises 7 questions, with a total score of 14 points, where 12‐14 points indicate normal nutritional status, a score of 8‐11 points suggests a risk of malnutrition, and a score below 8 indicates malnutrition. In this study, nutritional status was categorized into 2 groups: normal (12‐14 points) and at risk of malnutrition (11 points or below).

### Statistical Analyses

Distribution of the characteristics of the study participants is shown in Table 1. We examined the associations between PhAs (whole-body and segmental) and cognitive functions (overall and subdomains) in the older Korean population using multiple linear regression models after adjusting for all covariates. Associations were estimated using unstandardized coefficients (*b*) and 95% CIs. All analyses were conducted using SPSS software, version 27.0 (IBM).

**Table 1.** Categorical characteristics of older adults from a cross-sectional study conducted in Busan, South Korea, between July and December 2022, including age, gender, depressive symptoms, and nutritional status.

Categorical variables	Number of participants (N=625), n (%)
**Age (years)**	
65‐74	265 (42.4)
≥75	360 (57.6)
**Sex**	
Women	444 (71)
Men	181 (29)
**Depressive symptoms**	
Normal	421 (67.4)
At risk for depression	204 (32.6)
**Nutritional status**	
Normal	450 (72)
At risk of malnutrition	175 (28)

### Ethical Considerations

This study was approved by the Institutional Review Board of the Pusan National Hospital (2206-016-116), and written informed consent was obtained from all participants before the survey was administered. All methods adhered to the Declaration of Helsinki. Study data were anonymized to protect participants’ privacy. No compensation was provided to participants.

## Results

A total of 625 participants were analyzed. Tables1  2 list the participants’ characteristics. The participants had an average age of 76.0 years (SD 6.0), and 444 (71%) were women. More than half of the participants were not at risk of depression (n=421, 67.4%) and 450 out of 625 participants had normal nutritional status (72%). The average BMI was 24.5 kg/m²; the average whole-body PhA was 5.3 degrees, with the PhA of the upper limbs at 5.1 degrees, the PhA of the lower limbs at 5.4 degrees, and trunk PhA at 5.8 degrees. The overall cognitive function scores, along with domain-specific scores such as orientation, attention and calculation, memory, language, verbal comprehension and functional capacity, and constructional praxis, are presented in Table 2.

**Table 2.** Continuous characteristics of older adults (N=625) from a cross-sectional study in Busan, South Korea (July to December 2022), including age, BMI, cognitive function scores, and segmental PhAs^a^.

Continuous variables	Quantity, mean (SD)
Age (years)	76.0 (6.0)
BMI (kg/m^2^)	24.5 (3.5)
**Cognitive function scores**	24.7 (4.5)
Orientation scores	9.0 (1.8)
Attention and calculation scores	5.7 (2.0)
Memory scores	1.9 (1.0)
Language scores	4.7 (0.7)
Verbal comprehension and functional capacity scores	2.7 (0.6)
Constructional praxis scores	0.7 (0.5)
**PhA (degrees)**	
Upper-body PhA	5.1 (0.8)
Lower-body PhA	5.4 (1.1)
Trunk PhA	5.8 (5.9)
Whole-body PhA	5.3 (0.8)

a PhA: phase angle

Table 3 demonstrates a positive association between the whole-body PhA and overall cognitive function (*b*=0.62, 95% CI 0.16-1.08; *P*=.009) as well as some subdomains of cognitive function, such as orientation (*b*=0.31, 95% CI 0.13-0.50; *P*=.001) and memory (*b*=0.11, 95% CI 0.00-0.22; *P*=.04). Positive associations with cognitive functions—overall and all subdomains, with the exception of verbal comprehension, functional capacity, and constructional praxis—were mainly found for the PhA of the lower limbs. A positive association was also observed between the PhA of the upper limbs and orientation. No association was found between the PhA of the trunk and cognitive function.

**Table 3.** Multiple linear regression model examining associations between whole-body and segmental PhAs^a^ with cognitive function domains in older adults (N=625) from Busan, South Korea, between July and December 2022, adjusting for gender, age, BMI, the 15-item Geriatric Depression Scale total score, and nutritional status.

Cognitive function	Whole-body PhA			Upper-body PhA			Lower-body PhA			Trunk PhA		
	*b*	95% CI	*P* value	*b*	95% CI	*P* value	*b*	95% CI	*P* value	*b*	95% CI	*P* value
Overall	0.62	0.16 to 1.08	.009	0.39	−0.11 to 0.90	.13	0.72	0.38 to 1.06	<.001	0.01	−0.05 to 0.06	.80
Orientation	0.31	0.13 to 0.50	.001	0.29	0.09 to 0.49	.006	0.24	0.10 to 0.38	.001	−0.00	−0.02 to 0.02	.96
Attention and calculation	0.11	−0.10 to 0.32	.30	−0.03	−0.26 to 0.20	.79	0.21	0.06 to 0.37	.007	0.01	−0.02 to 0.03	.64
Memory	0.11	0.00 to 0.22	.04	0.04	−0.08 to 0.16	.47	0.14	0.05 to 0.22	.001	0.00	−0.01 to 0.02	.86
Language	0.01	−0.06 to 0.08	.80	−0.02	−0.09 to 0.06	.62	0.07	0.01 to 0.12	.014	0.00	−0.01 to 0.01	.93
Verbal comprehension and functional capacity	0.07	−0.00 to 0.14	.06	0.07	−0.00 to 0.15	.05	0.04	−0.01 to 0.09	.11	0.00	−0.01 to 0.01	.58
Constructional praxis	−0.01	−0.06 to 0.05	.84	−0.05	−0.10 to 0.01	.09	0.04	−0.00 to 0.07	.06	0.00	−0.00 to 0.01	.50

a PhA: phase angle.

## Discussion

### Principal Findings

This study is among the first to analyze the association between PhA and cognitive function in older adults. Positive associations were found between PhAs (whole-body and segmental) and cognitive function (overall and subdomains). Positive associations were found mainly between the PhA of the lower limbs and the orientation domain of cognitive function.

Our data show a positive association between whole-body PhA and overall cognitive function in an older population. A previous study of 153 patients with Alzheimer disease or amnestic mild cognitive impairment from Japan showed that decreased PhAs were associated with deteriorated cognitive function [19]. The positive association between whole-body PhA and overall cognitive function may be attributed to the integrity of neural cell functions and cellular health [7]. Cognitive decline with neural cell functional impairment and increased levels of oxidative stress are stress markers [34] in older adults. Previous studies have confirmed that PhAs can serve as a screening tool for oxidative damage because lower PhA values are related to higher levels of inflammatory markers, such as C-reactive protein, interleukin-6, and interleukin-10 [8,35]. Such positive associations were not only found for patients in the previous study but also for the generally healthy older population in this study. Another interesting finding in this study was the highly significant association between PhA and orientation and memory, all of which are related to the hippocampus. However, the specific mechanisms underlying this association require further investigations.

Our analyses showed that the PhA of the lower limbs plays a significant role among the PhAs for different body segments. Lower-limb PhA was positively associated with overall cognitive function and all its subdomains, except for verbal comprehension, functional capacity, and constructional praxis. This association likely stems from the relationship between lower-limb PhA and limb strength in older adults [36], which affects cognitive function by improving blood flow and reducing inflammation [37,38]. These mechanisms are crucial for maintaining brain health and cognitive performance. While additional evidence is needed to better understand the mechanisms underlying lower-limb PhA’s relationship with different cognitive dimensions, an earlier study has suggested that aerobic walking activities that involve lower-limb strength may contribute to increasing the size of the anterior hippocampus in older adults, thereby enhancing spatial memory and reducing the risk of cognitive decline [39]. In contrast, the PhA of the upper limbs showed a positive association with orientation. An interventional study of 31 older adults aged 65 years and above with mild cognitive impairment showed that an 8-week calligraphy treatment, an activity requiring upper-limb dexterity, could lead to significant improvements in some specific dimensions of cognitive function, including orientation and attention and calculation after 2 months, compared with their counterparts [40]. However, trunk PhA did not exhibit a significant relationship with any cognitive function, hinting at its potentially limited role in cognitive health. Further research, bolstered by additional evidence, is warranted to elucidate the underlying mechanisms.

This study has some limitations. First, this study was cross-sectional and the investigated associations between PhAs and cognitive function cannot be inferred as causal relationships. Future studies could benefit from adopting a longitudinal design to monitor cognitive function changes over time. Second, participants’ level of cognitive functioning was self-reported, which may lead to recall bias. Additionally, the predictive capability of the MMSE for cognitive decline varies, with studies showing that it might not effectively track early cognitive changes, particularly in comparison with more detailed assessments, such as the Montreal Cognitive Assessment (MoCA) [41]. We did not use cognitive functioning measured by MoCA, since data were unavailable; future research is suggested to incorporate the MoCA in the measurements of cognitive functioning. Third, some residual covariates, such as socioeconomic status, may not have been considered in the association between PhAs and cognitive function. Finally, participants of this study may be at different levels of frailty. Future studies should not only incorporate additional potential confounding variables for a more comprehensive analysis but also conduct more detailed subgroup analyses based on participants’ varying health conditions.

### Conclusions

Our results strengthen the limited evidence in the literature indicating that whole-body PhA is positively associated with cognitive function in older populations. Segmental PhAs reflect differential associations with cognitive function, highlighting the intricate relationship between body composition and cognitive health. These findings provide clinical implications on the potential strategies of primary screening of cognitive impairment among older adults by identifying those with a low PhA. Future research is suggested to further investigate the mechanisms and effectiveness of programs on strengthening physical function in different body regions on cognitive function in older adults.

## Abbreviations

BIA: bioelectrical impedance analyzer
BUSAN: Bus-based Screening and Assessment Network
K-MMSE: Korean version of the Mini-Mental State Examination
MoCA: Montreal Cognitive Assessment
PhA: phase angle

## References

[1] Murman DL (2015). The impact of age on cognition. Semin Hear.

[2] Gardner RC, Valcour V, Yaffe K (2013). Dementia in the oldest old: a multi-factorial and growing public health issue. Alzheimers Res Ther.

[3] Doroszkiewicz H (2022). How the cognitive status of older people affects their care dependency level and needs: a cross-sectional study. Int J Environ Res Public Health.

[4] Bu Z, Huang A, Xue M, Li Q, Bai Y, Xu G (2021). Cognitive frailty as a predictor of adverse outcomes among older adults: a systematic review and meta-analysis. Brain Behav.

[5] (2019). Risk reduction of cognitive decline and dementia: WHO guidelines. World Health Organization.

[6] Tan BL, Norhaizan ME (2021). The Role of Antioxidants in Longevity and Age-Related Diseases.

[7] Norman K, Stobäus N, Pirlich M, Bosy-Westphal A (2012). Bioelectrical phase angle and impedance vector analysis--clinical relevance and applicability of impedance parameters. Clin Nutr.

[8] da Silva BR, Orsso CE, Gonzalez MC (2023). Phase angle and cellular health: inflammation and oxidative damage. Rev Endocr Metab Disord.

[9] Hatanaka S, Shida T, Osuka Y (2024). Association between phase angle and inflammatory blood biomarkers in community-dwelling older adults: Itabashi longitudinal study on aging. Clin Nutr ESPEN.

[10] Reljic D, Zarafat D, Jensen B (2020). Phase angle and vector analysis from multifrequency segmental bioelectrical impedance analysis: new reference data for older adults. J Physiol Pharmacol.

[11] Wu H, Ding P, Wu J, Yang P, Tian Y, Zhao Q (2022). Phase angle derived from bioelectrical impedance analysis as a marker for predicting sarcopenia. Front Nutr.

[12] Norman K, Herpich C, Müller-Werdan U (2023). Role of phase angle in older adults with focus on the geriatric syndromes sarcopenia and frailty. Rev Endocr Metab Disord.

[13] Uemura K, Doi T, Tsutsumimoto K (2020). Predictivity of bioimpedance phase angle for incident disability in older adults. J Cachexia Sarcopenia Muscle.

[14] Asano Y, Tsuji T, Kim M (2023). Cross‐sectional and longitudinal study of the relationship between phase angle and physical function in older adults. Geriatr Gerontol Int.

[15] Sterner DA, Stout JR, Lafontant K, Park JH, Fukuda DH, Thiamwong L (2024). Phase angle and impedance ratio as indicators of physical function and fear of falling in older adult women: cross-sectional analysis. JMIR Aging.

[16] Sui SX, Williams LJ, Holloway-Kew KL, Hyde NK, Leach S, Pasco JA (2022). Associations between muscle quality and cognitive function in older men: cross-sectional data from the geelong osteoporosis study. J Clin Densitom.

[17] Sui SX, Williams LJ, Holloway-Kew KL, Hyde NK, Pasco JA (2020). Skeletal muscle health and cognitive function: a narrative review. Int J Mol Sci.

[18] Bellido D, García-García C, Talluri A, Lukaski HC, García-Almeida JM (2023). Future lines of research on phase angle: strengths and limitations. Rev Endocr Metab Disord.

[19] Yamada Y, Watanabe K, Fujisawa C (2024). Relationship between cognitive function and phase angle measured with a bioelectrical impedance system. Eur Geriatr Med.

[20] Lukaski HC, Garcia-Almeida JM (2023). Phase angle in applications of bioimpedance in health and disease. Rev Endocr Metab Disord.

[21] Tanaka S, Ando K, Kobayashi K (2019). The decreasing phase angles of the entire body and trunk during bioelectrical impedance analysis are related to locomotive syndrome. J Orthop Sci.

[22] Jun MH, Kim S, Ku B (2018). Glucose-independent segmental phase angles from multi-frequency bioimpedance analysis to discriminate diabetes mellitus. Sci Rep.

[23] Hurt RT, Ebbert JO, Croghan I (2021). The comparison of segmental multifrequency bioelectrical impedance analysis and dual-energy x-ray absorptiometry for estimating fat free mass and percentage body fat in an ambulatory population. J Parenter Enteral Nutr.

[24] Ng BK, Liu YE, Wang W (2018). Validation of rapid 4-component body composition assessment with the use of dual-energy x-ray absorptiometry and bioelectrical impedance analysis. Am J Clin Nutr.

[25] Yanishi M, Kinoshita H, Tsukaguchi H (2018). Dual energy x-ray absorptiometry and bioimpedance analysis are clinically useful for measuring muscle mass in kidney transplant recipients with sarcopenia. Transplant Proc.

[26] Fu L, Ren Z, Liu X (2022). Reference data of phase angle using bioelectrical impedance analysis in overweight and obese Chinese. Front Endocrinol (Lausanne).

[27] Uemura K, Yamada M, Okamoto H (2019). Association of bioimpedance phase angle and prospective falls in older adults. Geriatr Gerontol Int.

[28] Kang Y, Na DL, Hahn S (1997). A validity study on the Korean Mini-Mental State Examination (K-MMSE) in dementia patients. J Korean Neuro Assoc.

[29] Jeong SK, Cho KH, Kim JM (2004). The usefulness of the Korean version of modified Mini-Mental State Examination (K-mMMSE) for dementia screening in community dwelling elderly people. BMC Public Health.

[30] Kim R, Kim HJ, Kim A, Jang MH, Kim HJ, Jeon B (2018). Validation of the conversion between the Mini-Mental State Examination and Montreal Cognitive assessment in Korean patients with Parkinson disease. J Mov Disord.

[31] Sheikh JI, Yesavage JA (1986). Geriatric Depression Scale (GDS): recent evidence and development of a shorter version. Clin Gerontol.

[32] Shin C, Park MH, Lee SH (2019). Usefulness of the 15-item geriatric depression scale (GDS-15) for classifying minor and major depressive disorders among community-dwelling elders. J Affect Disord.

[33] Guigoz Y, Vellas B (2021). Nutritional assessment in older adults : MNA® 25 years of a screening tool and a reference standard for care and research; what next?. J Nutr Health Aging.

[34] Kandlur A, Satyamoorthy K, Gangadharan G (2020). Oxidative stress in cognitive and epigenetic aging: a retrospective glance. Front Mol Neurosci.

[35] Tomeleri CM, Cavaglieri CR, de Souza MF (2018). Phase angle is related with inflammatory and oxidative stress biomarkers in older women. Exp Gerontol.

[36] Homma D, Minato I, Imai N (2023). Associations of lower-limb phase angle with locomotion and motor function in Japanese community-dwelling older adults. Geriatrics (Basel).

[37] Kong J, Kang M, Kang H (2022). The relationship between late-life depression and cognitive function in older Korean adults: a moderation analysis of physical activity combined with lower-body muscle strength. Int J Environ Res Public Health.

[38] Herold F, Törpel A, Schega L, Müller NG (2019). Functional and/or structural brain changes in response to resistance exercises and resistance training lead to cognitive improvements - a systematic review. Eur Rev Aging Phys Act.

[39] Erickson KI, Voss MW, Prakash RS (2011). Exercise training increases size of hippocampus and improves memory. Proc Natl Acad Sci USA.

[40] Kwok TCY, Bai X, Kao HSR, Li JCY, Ho FKY (2011). Cognitive effects of calligraphy therapy for older people: a randomized controlled trial in Hong Kong. Clin Interv Aging.

[41] Mitchell AJ, Larner AJ (2017). Cognitive Screening Instruments: A Practical Approach.

